# Analysis of a clinical process of schizophrenia and other psychoses with a process mining method

**DOI:** 10.1192/j.eurpsy.2021.1071

**Published:** 2021-08-13

**Authors:** E. Rodríguez Toscano, B. Reneses, S. Pérez, P. Andrés, G. Seara, J. Sevilla, A. Cortés, Á. Del Rey-Mejías

**Affiliations:** Institute Of Psychiatry And Mental Health, San Carlos University Hospital, Madrid, Spain

**Keywords:** Clinical stage, schizophrénia, Clinical pathways, Process mining method

## Abstract

**Introduction:**

Clinical pathways (CPWs) are tools used to guide evidence-based healthcare. They translate clinical practice guideline recommendations into clinical processes of care within the characteristics of a healthcare institution. There are few studies about the impact of CPW in the field of Psychosis in terms of adequacy to their recommendations and clinical outcomes.

**Objectives:**

PSYCHSTAGE project has been designed to study the adjustment of psychosis clinical care to a CPW based in a Clinical Practice Guideline according to a clinical staging model in a network of psychiatric services covering 580.000 inhabitants in a University Hospital in Madrid.

**Methods:**

Retrospective and observational study in a sample of 1780 subjects 18 years old or above, diagnosed with schizophrenia and other psychosis. Socio-demographic and clinical variables were collected from clinical records, including ICG, GAF and DAS at the time they were included in the study. Clinical stage was established according to McGorry model at the same time. CPW was analysed in 1,391 subjects with 15,254 care events using a Process mining method. Process discovery, process checking and process enhancement analysis have been used.

**Results:**

Patients were grouped according the clinical stage. 9,2% were in stage 2; 18,5% in 3a; 47% in 3b; 22,1% in 3c and 4,1% in 4. A different CPW is represented for each clinical stage in routine practice. Then, every pathway is compared with the recommendations in the established Psychosis CPW.

**Conclusions:**

Process mining can be a useful tool for the study of CPW in the field of psychosis

